# Neonatal meningitis, endocarditis, and pneumonitis due to *Streptococcus gallolyticus subsp. pasteurianus*: a case report

**DOI:** 10.1186/s12887-019-1645-x

**Published:** 2019-08-01

**Authors:** Michelle T. Nguyen, Soha Idriss, Ernie Guzman, Elizabeth R. De Oliveira

**Affiliations:** 1 Department of Obstetrics and Gynecology, White Memorial Medical Center, 1720 Cesar E. Chavez Avenue, Los Angeles, CA 90033 USA; 2 Department of Neonatology, White Memorial Medical Center, Los Angeles, CA 90033 USA; 3 Department of Pediatric Infectious Diseases, White Memorial Medical Center, Los Angeles, CA 90033 USA; 4 Pacific Pediatric Cardiology Medical Group, Inc., Los Angeles, CA 90033 USA

**Keywords:** Sepsis, Meningitis, Endocarditis, Pneumonitis, Meconium, *Streptococcus pasteurianus*

## Abstract

**Background:**

*Streptococcus pasteurianus* is a rare cause of neonatal infection, with only 3 cases reported in the USA and 18 cases reported in other countries within the past decade. Neonatal *S. pasteurianus* infection typically presents as meningitis. This case report describes the first neonatal case of *S. pasteurianus* endocarditis in the literature, in addition to a neonatal case of *S. pasteurianus* infection presenting as pneumonitis without meningitis. The *S. pasteurianus* infections in these two cases are unusual not only because of how rare this particular pathogen is, but also because of the atypical clinical manifestations.

**Case presentation:**

The first patient is a full-term male infant admitted to NICU at 20 h of life due to respiratory distress. He was empirically started on ampicillin and gentamicin for presumed sepsis. Laboratory analysis of cerebral spinal fluid obtained after initiation of antibiotics was suggestive of partially treated meningitis. Blood cultures came back positive for *S. pasteurianus*. The neonate was transitioned from ampicillin to cefepime, while gentamicin was continued. Echocardiograph showed a possible tricuspid valve vegetation concerning for endocarditis. Due to the unusual complication of endocarditis, the patient remained on IV cefepime for 28 days rather than the more conventional duration of 14–21 days reported in the literature. The baby clinically improved with no evidence of thrombi or vegetations on repeat cardiac echo.

The second patient is a full-term male infant who required intubation at birth for respiratory distress. Chest X-ray findings were concerning for meconium aspiration with pneumonitis. The baby went into septic shock and was empirically started on ampicillin and gentamicin. Blood cultures came back positive for *S. pasteurianus*, while cerebral spinal fluid and urine cultures were negative. Ampicillin and gentamicin were discontinued after 3 days and the baby was started on cefepime and clindamycin for a total 14-day course. The baby clinically recovered and was discharged from NICU without any sequelae.

**Conclusions:**

These two cases highlight the importance of recognizing *S. pasteurianus* as a potential cause of neonatal sepsis and the importance of recognizing endocarditis and pneumonitis as possible clinical manifestations of this infection.

## Introduction

*Streptococcus gallolyticus subsp. pasteurianus*, also known as *Streptococcus pasteurianus* or *Streptococcus bovis* biotype II/2, is a relatively rare cause of neonatal infection, with only three cases reported in the USA within the past decade [1–3]. When neonates are infected with this pathogen, the clinical presentation typically manifests as sepsis with meningitis, similar to that of group B *streptococcus* (GBS) infection. We present two cases of *S. pasteurianus* infection in neonates who were admitted to NICU within the first 24 h of life due to respiratory distress attributed to meconium aspiration syndrome. One infant developed sepsis (bacteremia and meningitis) with endocarditis, which is the first reported case of neonatal endocarditis due to *S. pasteurianus*. The other infant developed sepsis and pneumonitis without meningitis.

## Case presentation

### Patient 1

**Fig. 1:**
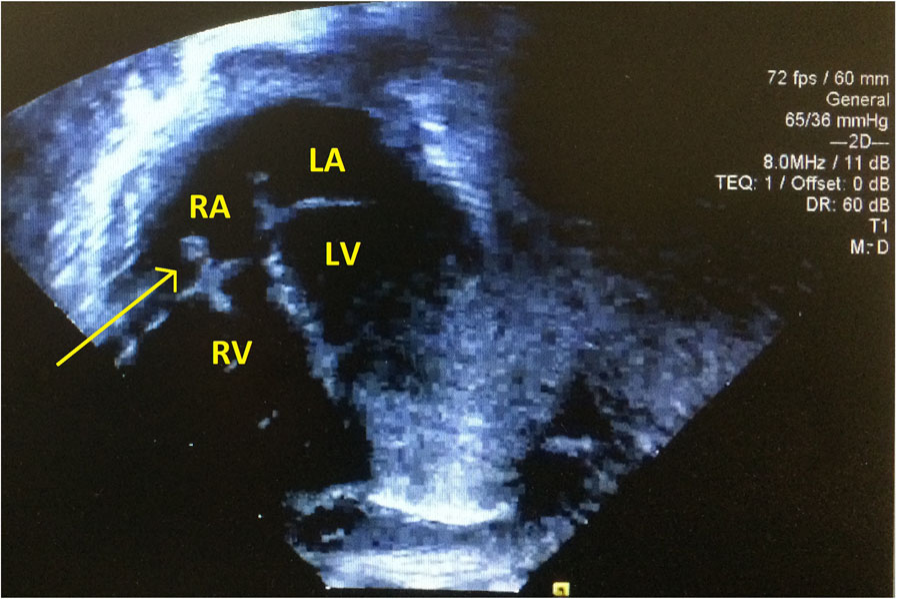
Apical 4-chamber view on echocardiograph showing a 0.40-cm vegetative lesion on the tricuspid valve (indicated by arrow). RA = right atrium, RV = right ventricle, LA = left atrium, LV = left ventricle

**Fig. 2:**
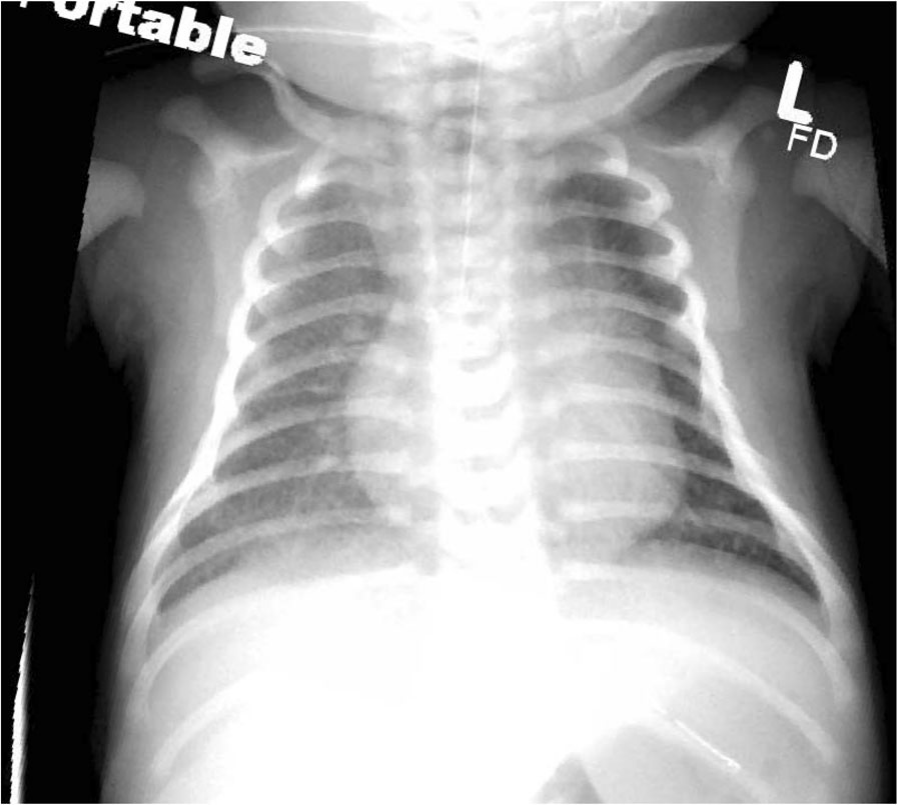
CXR for patient 1: mild diffuse pulmonary ground-glass changes are seen due to minimal reactive edema related to mild meconium aspiration. OGT projects with tip and side-hole below the left hemidiaphragm

A male infant weighing 4195 g was born at 39 weeks and 1 day to a GBS-negative mother via spontaneous vaginal delivery 12 h after artificial rupture of membranes with thick meconium. The infant was large for gestational age; the mother was obese with a BMI of 46.6 kg/m^2^ but tested negative for gestational diabetes. Apgar scores were 4 and 8 at 1 and 5 min, respectively. At birth the infant required stimulation and suctioning but recovered well and initially remained with mother. At 20 h of life, the infant was noted to be in respiratory distress with tachypnea, tachycardia, and O2 desaturations down to 85%; therefore, he was admitted to NICU with oxygen via nasal cannula. Blood and urine cultures were obtained and the infant was empirically started on ampicillin and gentamicin for presumed sepsis. Chest X-ray findings were suggestive of meconium aspiration (Fig. 1). Initial laboratory values were significant for a lactic acid of 7.4 mmol/L and a C-reactive protein (CRP) of 6.4 mg/dL. However, leukocyte count, hemoglobin, platelets, serum glucose, electrolytes, blood urea nitrogen, and creatinine were within normal limits. Cerebral spinal fluid (CSF) obtained 12 h after initiation of antibiotic therapy appeared hazy with WBC of 41 cell/mm^3^ (polymorphonuclear leukocytes of 67% and mononuclear cells 14%), RBC of 10 cell/mm^3^, glucose of 53 mg/dL, and protein of 76 mg/dL, suggestive of partially treated meningitis. Point of care blood glucose level at the time of lumbar puncture was 77. On day of life #4, blood cultures came back positive for *Streptococcus gallolyticus subsp. pasteurianus*; CSF and urine cultures were negative. While waiting for sensitivity results, the patient was transitioned from ampicillin to cefepime for broader coverage and ease of use, and gentamicin was continued. Due to the known association of *S. pasteurianus* with endocarditis and myocarditis in adults, Infectious Disease also recommended an echocardiograph, which was performed on day of life #4. Cardiology noted a possible vegetative lesion attached to the tricuspid valve apparatus on echo (Fig. 2), which was concerning for endocarditis. Therefore, the patient remained on gentamicin for 14 days and cefepime for 28 days. Blood cultures subsequently revealed sensitivity to ceftriaxone, clindamycin, penicillin G, and vancomycin. The patient clinically improved with resolution of tachypnea and transition to room air. Repeat echocardiographs performed at 1 and 3 weeks after the initial cardiac echo showed no evidence of thrombi or vegetations. The baby was discharged from NICU on day of life #31 without any sequelae.

### Patient 2

**Fig. 3:**
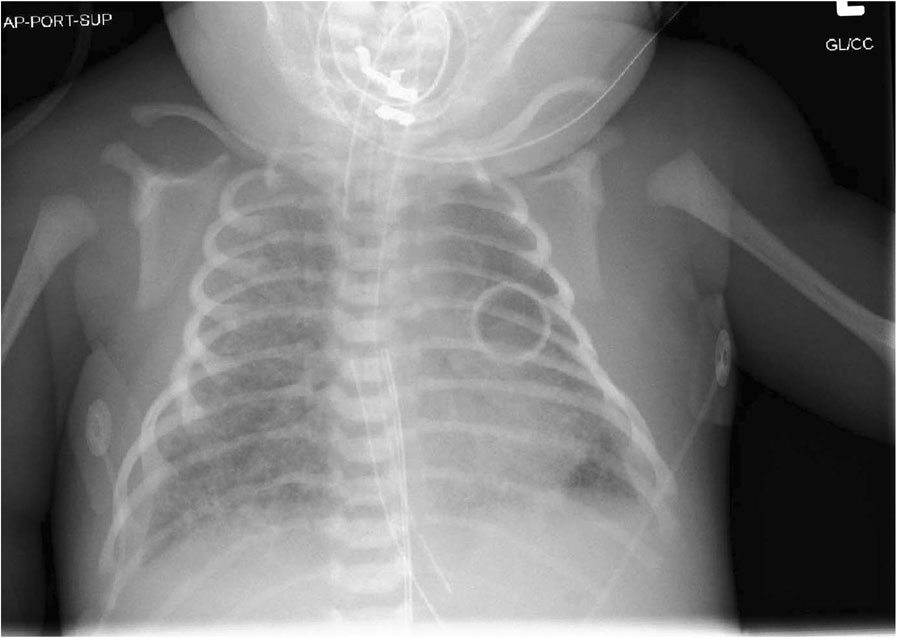
CXR for patient 2: hazy granularity involving the lung fields is present, in addition to patchy bilateral infiltrates with small pleural effusions. Endotracheal tube and umbilical catheter are in place

A male infant weighing 3250 g was born at 40 weeks and 1 day (appropriate for gestational age) to a GBS-unknown mother via spontaneous vaginal delivery 4 h after artificial rupture of membranes with thick meconium. No intrapartum antibiotics for GBS prophylaxis were given as membranes were ruptured for < 18 h. Apgar scores were 8 and 8 at 1 and 5 min, respectively. The infant was drowsy at birth and showed irritability and poor respiratory effort requiring CPAP, followed by bag-valve-mask ventilation while in transit to NICU. Upon arrival to NICU, patient was immediately intubated and given one dose of surfactant due to severe respiratory distress requiring FiO2 at 100%. Blood appeared from the endotracheal tube shortly after administration of surfactant, which was concerning for pulmonary hemorrhage as a complication of meconium aspiration. At that time persistent pulmonary hypertension of the newborn (PPHN) was also clinically suspected in the setting of meconium aspiration and pulmonary hemorrhage. Initial chest X-ray showed patchy bilateral infiltrates, which was concerning for pneumonitis as an additional complication of meconium aspiration (Fig. 3). The baby subsequently became hypotensive and went into septic shock requiring dopamine and dobutamine pressors. Cultures from blood, urine, and endotracheal tube aspirate were obtained and the infant was empirically started on ampicillin and gentamicin. Initial laboratory values were significant for 17,700 leukocytes/mm^3^ (14% band neutrophils, 25.3% segmented neutrophils, 50.5% lymphocytes, 7.4% monocytes, and 2.5% eosinophils) on complete blood cell count, lactic acid of 2.1 mmol/L, CRP of 11.4 mg/dL, and procalcitonin of 71.21 ng/mL. CSF was unremarkable with clear appearance, no WBC’s, glucose of 73 mg/dL, and protein of 66 mg/dL. Echocardiograph on day of life #2 revealed a flattened interventricular septum implying elevated right-sided pressures (consistent with pulmonary hypertension), but no evidence of thrombi or vegetations. On day of life #3, the baby’s blood cultures came back positive for *Streptococcus gallolyticus subsp. pasteurianus*; CSF, urine, and endotracheal tube aspirate cultures were negative. While waiting for sensitivity results, ampicillin and gentamicin were discontinued and the baby was started on cefepime (for broader coverage) and clindamycin (for antitoxin effect in the setting of septic shock) for a total 14-day course of antibiotics. Blood cultures subsequently revealed sensitivity to ceftriaxone, penicillin G, and vancomycin. The baby clinically recovered, was extubated on day of life #8, and was discharged from NICU on day of life #21 without any sequelae.

## Discussion

As of 2008, there were a total of 30 neonatal cases of *S. pasteurianus* sepsis in the English literature [4]. Since then, an additional 3 cases have been reported in the USA and 18 cases reported in other countries [5–9]. This does not include one case report of neonatal *S. bovis* meningitis in the USA in 2012, in which the subspecies was not identified [10]. The exact prevalence of neonatal infection with *S. pasteurianus* or other subspecies of *S. bovis* is difficult to determine due to the possibility of past cases being misidentified as other group D streptococci, especially before the currently available techniques for microbial gene sequencing. Group D streptococci include *Streptococcus bovis* in addition to *Streptococcus agalactolyticus*, *Streptococcus equinus*, and *Enterococcus* [10]. With new molecular analysis techniques leading to taxonomic changes, *Streptococcus bovis* has been further subdivided into *Streptococcus gallolyticus subsp. gallolyticus* (also known as *S. gallolyticus* or *S. bovis* biotype I), *Streptococcus gallolyticus subsp. infantarius* (also known as *S. infantarius* or *S. bovis* biotype II/1), and *Streptococcus gallolyticus subsp. pasteurianus (*also known as *S. pasteurianus* or *S. bovis* biotype II/2) [3].

The precise identification of *S. bovis* subspecies is important due to the different clinical implications of each subspecies. *S. gallolyticus* is associated with colonic malignancies and endocarditis in adults, *S. infantarius* with non-colonic malignancies in adults, and *S. pasteurianus* with sepsis and meningitis in both adults and neonates [7]. The clinical presentation of meconium aspiration syndrome or neonatal infection with *S. pasteurianus* strongly resembles that with other pathogens such as group B *streptococcus* (which we initially considered in our differential diagnosis when the preliminary blood cultures revealed Gram-positive cocci in pairs and chains), *Listeria monocytogenes*, and *Escherichia coli* [11]. The presentation of *S. pasteurianus* sepsis in our first patient was very unusual because the baby developed endocarditis. *Streptococcus bovis* endocarditis is more strongly associated with *S. gallolyticus*, not *S. pasteurianus*, and generally affects adults [7]. No cases of neonatal *Streptococcus bovis* endocarditis have ever been reported in the literature prior to our patient. Due to the complication of endocarditis, our patient remained on IV cefepime for 28 days rather than the more conventional duration of 14–21 days reported in the literature. The presentation of *S. pasteurianus* sepsis in our second patient was also atypical because the baby developed pneumonitis without meningitis.

The source and mechanism of neonatal *S. bovis* infection remains uncertain. *S. bovis* has been found as part of normal colonic and oral flora in healthy patients [12]. *S. bovis* has also been isolated from normal vaginal secretions [13]. In fact, Fikar and Levy previously described a case of *S. bovis* in vaginal and rectal cultures from a mother whose infant developed *S. bovis* meningitis [14]. Colonization of the vagina leading to ascending intrauterine infection is also a possibility, as evidenced by one case of *S. pasteurianus* intrauterine infection and postpartum bacteremia in a mother whose baby subsequently had *S. pasteurianus* in his ear and throat swabs but remained asymptomatic [7]. It is not known whether the mothers in our cases had *S. pasteurianus* in their vagina or rectum, but regardless, they had a lower risk of ascending intrauterine infection in the absence of prolonged rupture of membranes. Nevertheless, both babies had thick meconium upon artificial rupture of membranes and subsequently developed meconium aspiration syndrome.

## Conclusions

Neonatal infection with *S. pasteurianus* is relatively rare and tends to present as sepsis with meningitis, similar to how neonatal infection with the more commonly recognized pathogen GBS presents. However, as demonstrated in our two patient cases, endocarditis and pneumonitis should also be recognized as potential sequelae of neonatal *S. pasteurianus* sepsis.

## Data Availability

Data sharing is not applicable to this article as no datasets were generated or analyzed during the current study.

## Abbreviations

GBS: Group B *streptococcus*
*S. pasteurianus*: *Streptococcus gallolyticus subsp. pasteurianus* or *Streptococcus pasteurianus*
